# Duration of persistence of molluscum contagiosum: A retrospective study

**DOI:** 10.1016/j.jdin.2025.02.004

**Published:** 2025-04-09

**Authors:** Robin C. Yi, Dillon Razler, Morgan L. Agner, Lauren N. McGrath, Brett R. Shaffer, Zeynep M. Akkurt, Steven R. Feldman

**Affiliations:** a Center for Dermatology Research, Department of Dermatology, Wake Forest University School of Medicine, Winston-Salem, North Carolina; b Department of Pathology, Wake Forest University School of Medicine, Winston-Salem, North Carolina; c Department of Social Sciences & Health Policy, Wake Forest University School of Medicine, Winston-Salem, North Carolina

**Keywords:** average duration of molluscum contagiosum, molluscum contagiosum, sequalae after molluscum contagiosum, treatments for molluscum contagiosum

*To the Editor:* Molluscum contagiosum (MC) often require treatment due to cosmetic and transmission concerns. Limited studies investigated how long MC lesions persist for untreated patients and findings are conflicting.^1^, ^2^, ^3^, ^4^ This study compared the outcomes between untreated and treated MC cases.

A retrospective chart review and phone survey analyzed patients with MC from 2013 to 2023 at the Atrium Health Wake Forest Baptist. Data were collected on treatment, immunocompromised status, and sequalae. Statistical analysis compared categorical variables using *t*-tests and ANOVA.

A total of 252 participants diagnosed with MC had a mean age of 11.1 ± 5.6 years (Table I). 89 participants responded to the phone survey. The mean persistence without treatment was 9.9 ± 8.4 months, with 76.9% of participants reporting no sequalae and 23.1% reporting scarring or pigmentation alterations. The mean persistence with treatment was 8.5 months ± 2.6 months. There were no immunodeficiencies among participants.

**Table I:** Demographics of participants (*n* = 252) which include the sex, race, mean age in years, and total number of treated and untreated participants in 5-year age groupings

Characteristic	Value
Sex, *n* (%)	
Male	135 (53.6)
Female	117 (46.4)
Race, *n* (%)	
White	150 (59.5)
Black	50 (19.8)
Hispanic	47 (18.7)
Asian	1 (0.4)
Other	4 (1.6)
Mean age in y, *n* (range)	11.08 (2-24)
Ages 0-5 y, *n*	
Treated	0
Untreated	9
Ages 6-10 y, *n*	
Treated	10
Untreated	42
Ages 11-15 y, *n*	
Treated	10
Untreated	23
Ages 16-20 y, *n*	
Treated	2
Untreated	5
Ages 21-25 y, *n*	
Treated	1
Untreated	1

The untreated mean persistence of MC was stratified amongst 5-year age groups and ranged from 6.2 to 12.8 months, which was statistically similar between groups (*P* = .09). The untreated mean persistence between participants with reported scarring as sequelae versus those with no skin changes was not significantly different (*P* = .31). The treated mean persistence of MC ranged from 7 to 8.7 months in 5-year age groups, with no significant difference in mean persistence of MC between untreated (9.9 months) and treated participants (8.5 months, *P* = .16).

Kaplan–Meier analysis demonstrated a steeper decline in MC persistence among treated participants before 10 months (Fig 1). Treatment may prevent disease persistence beyond 1 year, though some untreated cases lasted up to 2 or 3 years.

**Fig 1:**
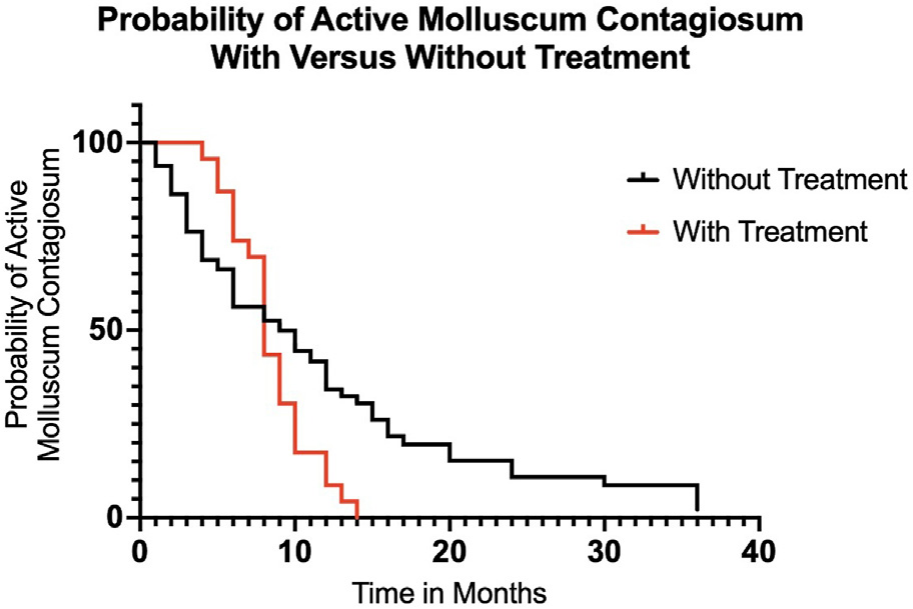
A Kaplan–Meier Curve depicts the probability of active molluscum contagiosum comparing treatment to those without treatment. Untreated patients may experience the persistence of disease for 2 or 3 years compared to treated patients with 1 year of disease persistence.

The mean age of patients was 11 years, slightly older than the typical MC onset of 6-7 years old.^5^ This may reflect delayed diagnosis, prolonged disease course, or sexual transmission in older age groups. The age diagnosed with MC did not significantly influence the natural progression of MC, as untreated mean persistence was similar across age groups (*P* = .09). While MC is more common in younger children, the overall disease duration remains relatively stable across different age groups.

The mean MC persistence without treatment was 9.9 months, shorter than the 13.3 months reported in prior studies.^1^ Most participants (77%) experienced resolution without long-term sequelae, and the presence of scarring or pigmentation changes did not correlate with prolonged disease duration (*P* = .31). These findings support that MC resolves without intervention, and persistent skin changes may be independent of infection duration.

Limitations include recall bias, lack of direct clinical observation, and exclusion of immunocompromised individuals. Unreported at-home remedies could have influenced persistence. Our findings reinforce that MC is self-limiting, and treatment decisions should be based on symptom burden and patient preference. Treatment modalities have a low incidence of scarring, which offers a solution for patients with a high burden of disease without complications.^5^ While local mechanical modalities can effectively remove lesions, treatment did not significantly shorten MC persistence and did not necessarily accelerate overall disease resolution. There remains a need for more effective treatments that can reduce MC persistence.

## Conflicts of interest

Dr Feldman has received research, speaking and/or consulting support from Eli Lilly and Company, GlaxoSmithKline/Stiefel, AbbVie, Janssen, Alovtech, vTv Therapeutics, Bristol-Myers Squibb, Samsung, Pfizer, Boehringer Ingelheim, Amgen, Dermavant, Arcutis, Novartis, Novan, UCB, Helsinn, Sun Pharma, Almirall, Galderma, Leo Pharma, Mylan, Celgene, Ortho Dermatology, Menlo, Merck& Co, Qurient, Forte, Arena, Biocon, Accordant, Argenx, Sanofi, Regeneron, the National Biological Corporation, Caremark, Teladoc, BMS, Ono, Micreos, Eurofins, Informa, UpToDate ,and the National Psoriasis Foundation. He is founder and part owner of Causa Research and holds stock in Sensal Health. Authors Yi, Razler, Agner, McGrath, Shaffer, and Dr Akkurt have no conflicts of interest to declare.
